# Synergising latent profile analysis and network analysis to elucidate the relationships among emotion dysregulation, post-traumatic stress disorder, dissociation, psychosis, and affective symptoms in individuals with trauma and severe mental illness

**DOI:** 10.1016/j.ijchp.2026.100714

**Published:** 2026-08-25

**Authors:** Jianlin Liu, Celeste Minn Tan, Adela-Maria Isvoranu, Sacha Epskamp, Yoke Boon Tan, Rachel Hsiao Shen Tan, Sherilyn Shi Hui Chang, Vanessa Ai Ling Seet, Phern Chern Tor, Yu Wei Lee, Charmaine Tang, Nisha Chandwani, Swapna Verma, Mythily Subramaniam

**Affiliations:** a Research Division, Institute of Mental Health, Singapore; b Department of Psychology, National University of Singapore, Singapore; c Department of Psychological Medicine, National University of Singapore, Singapore; d Department of Mood & Anxiety, Institute of Mental Health, Singapore; e Department of Psychosis, Institute of Mental Health, Singapore; f Duke-NUS Medical School, Singapore

**Keywords:** Trauma exposure, Dissociation, PTSD, Psychosis, Transdiagnostic

## Abstract

**Background:**

Trauma exposure and emotion dysregulation are transdiagnostic risk factors for post-traumatic stress disorder (PTSD) and severe mental disorders (e.g., depression, and schizophrenia spectrum disorders). The present study elucidated the latent profiles and interrelationships between emotion dysregulation and psychopathology (PTSD, dissociation, psychosis, and affective symptoms) in a trauma-exposed severe mental illness population.

**Methods:**

We recruited a large sample of trauma-exposed outpatients with mood disorders and schizophrenia spectrum disorders in Singapore (N = 461; M*_age_* = 35.47). PTSD and dissociation were assessed with the Clinician Administered PTSD Scale for DSM-5. Psychosis and affective symptoms were assessed with the Brief Psychiatric Rating Scale-Expanded. Emotion regulation ability deficits were assessed with the Difficulties with Emotion Regulation Scale. Maladaptive emotion regulation strategies were assessed with the Ruminative Response Scale (rumination), Penn State Worry Questionnaire (worry), and Emotion Regulation Questionnaire (expressive suppression).

**Results:**

Latent profile analysis identified three distinct risk profiles: severe emotion dysregulation and psychopathology (Profile 3; 19.1 %), moderate emotion dysregulation and psychopathology (Profile 2; 37.5 %), and low emotion dysregulation and psychopathology (Profile 1; 43.4 %). Network analysis identified distinct emotion dysregulation and psychopathology networks for each latent profile. Profile 3 had the highest network density and the strongest bridge connections between emotion dysregulation (poor emotional clarity) and psychopathology (PTSD symptoms of negative alterations in cognitions and mood and positive psychotic symptoms).

**Conclusions:**

We synergised both latent profile analysis and network analysis to identify subgroups of at-risk patients and elucidate interrelationships between emotion dysregulation and psychopathology within each subgroup to inform personalized assessments and interventions.

## Introduction

Trauma exposure is associated with post-traumatic stress disorder (PTSD) and a myriad of severe mental disorders, including but not limited to depression, bipolar disorder, dissociative disorders, and schizophrenia spectrum disorders (Gibson et al., 2016; McKay et al., 2021; Rnic et al., 2023; Sideli et al., 2020). Severe mental disorders are defined as chronic psychiatric conditions that require long-term treatment (Grubaugh et al., 2011; Halstead et al., 2024; Zumstein & Reise, 2020). While trauma exposure is required to establish a PTSD diagnosis (American Psychiatric Association, 2022), accumulating evidence shows strong associations between trauma/PTSD and severe mental disorders; hence, severe mental disorders are also considered trauma-related mental disorders (Ford, 2025; Goh et al., 2026; Liu et al., 2025; Wang et al., 2023).

A longstanding challenge in the field is the complexity of comorbid and overlapping symptoms of PTSD and trauma-related mental disorders (Grubaugh et al., 2011; Hansen et al., 2017; Klein et al., 2021). Traumatic flashbacks in PTSD overlap considerably with hallucinations in psychosis due to the dissociative nature of these symptoms. Negative mood and dysfunctional beliefs are also common across depression, psychosis, and PTSD. To circumvent these pertinent clinical issues, a transdiagnostic approach is proposed to inform assessment and interventions for PTSD and trauma-related mental disorders (Dalgleish et al., 2020; Stanton et al., 2020). Among the range of transdiagnostic psychological factors, converging research evidence points to emotion dysregulation as a pertinent transdiagnostic risk factor for PTSD and trauma-related mental disorders (Hales et al., 2023; Lynch et al., 2021; Miu et al., 2022).

Emotion dysregulation is a multidimensional construct (Naragon-Gainey et al., 2017; Tull et al., 2020). The strategy-based model conceptualizes emotion dysregulation as the habitual use of adaptive/maladaptive emotion regulation strategies; putatively maladaptive emotion regulation strategies are positively associated with long-term psychopathology, while putatively adaptive emotion regulation strategies are negatively associated with long-term psychopathology (Aldao et al., 2010; Naragon-Gainey et al., 2017; Pugach & Wisco, 2023; Seligowski et al., 2015). In contrast to the strategy-based model, the ability-based model conceptualizes emotion dysregulation as the lack of broad abilities to support healthy emotion regulation (Gratz & Roemer, 2004; Seligowski et al., 2015). These broad abilities correspond to the six scale dimensions of the Difficulties in Emotion Regulation Scale (i.e., poor emotional awareness, poor emotional clarity, impulsivity, non-acceptance of emotions, difficulties engaging in goal-directed behaviour, and limited access to adaptive emotion regulation strategies). Any of these emotion regulation ability deficits may lead to emotion dysregulation and psychopathology (Gratz & Roemer, 2004).

Theoretical and empirical evidence supports the integration of both the strategy-based and ability-based models due to their bidirectional effects. Emotion regulation ability deficits contribute to inflexible and inefficient use of emotion regulation strategies to meet specific individual goals and contextual demands (Tull et al., 2020). On the other hand, habitual use of putatively maladaptive emotion regulation strategies (e.g., experiential avoidance and expressive suppression of negative emotions) may negatively reinforce emotion regulation abilities (e.g., non-acceptance of negative emotions) (Tull et al., 2020). The bidirectional effects are supported by the Functional Model of Emotional Disorders underlying the Unified Protocol (UP) for transdiagnostic treatment of emotional disorders (Barlow, 2018), which postulates that over-reliance on avoidance-oriented emotion regulation strategies prevents learning of corrective information and reinforces beliefs that negative emotions are dangerous and unacceptable. Consequently, this creates a vicious cycle of avoidance and persistent negative emotions that contributes to the development and exacerbation of PTSD and other emotional disorders (Barlow, 2018). Hence, the integration of both strategy-based and ability-based models of emotion dysregulation supports a more complete understanding of the relationships among emotion dysregulation, PTSD, and trauma-related mental disorders (Liu et al., 2020a; Miu et al., 2022; Tull et al., 2020).

The relationships among emotion dysregulation, PTSD, and trauma-related mental disorders may be further understood through the integration of the developmental trauma (DT) framework and the conservation of resources (COR) theory. DT involves exposure to prolonged and complex life-threatening and stressful events during sensitive periods of socio-emotional development in childhood. Specifically, DT disrupts secure attachments with caregivers, distorts internal working models, alters core assumptions of safety, self-esteem, and perceived control, and weakens individual capacities for effective distress coping; hence, emotion dysregulation is a consequence of disturbed socio-emotional development due to early and prolonged trauma (Cruz et al., 2022). The DT framework also postulates that individuals may develop symptoms beyond PTSD (e.g., disturbances in self-organisation symptoms in complex PTSD and multiple trauma-related mental disorders) and symptom heterogeneity is attributed to individual differences (e.g., varying emotion regulation abilities), trauma characteristics, and environmental support (Cruz et al., 2022). Complementing the DT framework, the COR theory proposes that trauma results in resource loss (e.g., reduced emotion regulation capacities) that could mitigate the deleterious effects of trauma (Hobfoll, 2011). Further, the COR theory extends the DT framework to account for cumulative trauma exposure from childhood to adulthood (e.g., adverse childhood experiences and interpersonal partner violence) and the compounding depletion of emotion regulation resources in vulnerable individuals (Hobfoll, 2011; Simpson et al., 2025). COR theory provides a theoretical foundation for emotion dysregulation-focused treatments, such as Skills Training in Affective and Interpersonal Regulation (STAIR), where STAIR is focused on replenishing emotion regulation and interpersonal skills in trauma-exposed individuals (Cloitre et al., 2010; Corrigan et al., 2020).

Recent research findings have strengthened the theoretical links among emotion dysregulation, PTSD, and trauma-related mental disorders. A longitudinal study found that cumulative experiences of intimate partner violence predicted more severe emotion regulation ability deficits over time in young adult community women (Simpson et al., 2025). A systematic review of longitudinal and cross-sectional mediation studies reported converging evidence that emotion dysregulation mediated the association between psychological maltreatment and psychopathology (Schaumburg & Nimtz, 2026). A meta-analysis found a large effect size of childhood trauma on later emotion dysregulation (Standardized Mean Difference [SMD] = 1.25, 95 % Confidence Interval [CI]: [1.12, 3.62]) (Fan & Kang, 2025). Recent studies using ecological momentary assessments (EMA) observed that PTSD predicted the use of maladaptive emotion regulation strategies equally among European and Chinese Australian trauma survivors (Qiu et al., 2025) and emotion regulation flexibility was associated with lower momentary transdiagnostic psychopathology (depression, anxiety, and stress) among trauma-exposed veterans (Chen et al., 2026). Finally, new findings have surfaced on the association between emotion dysregulation and understudied trauma-related mental disorders, such as personality disorders and dissociative disorders (Ford, 2025; Thornton et al., 2025), and neurobiological underpinnings of emotion dysregulation in trauma-related mental disorders have also been elucidated (Jannini et al., 2025).

Previous research has predominantly investigated the associations between emotion dysregulation and psychopathology based on regression and factor analyses, which cannot determine heterogenous subgroups or account for interrelationships between emotion dysregulation and psychopathology. Given that individual differences contribute to heterogenous profiles of emotion dysregulation and psychopathology (as aligned with the DT framework) and emotion dysregulation and psychopathology may co-occur and reinforce each other (Dieujuste et al., 2025; Wang & Contractor, 2025), latent profile analysis (LPA) and network analysis are increasingly used to elucidate the complex relationships between emotion dysregulation and psychopathology. LPA aims to identify subgroups of individuals with varying risk profiles based on the endorsement patterns of key indicator variables. LPA research has identified subgroups with varying profiles of emotion regulation strategies (Pugach & Wisco, 2023), emotion regulation ability deficits (Garofalo et al., 2020), and PTSD subtypes (Hansen et al., 2017). Overall, findings from LPA research have identified subgroups of at-risk patients and personalized interventions can be provided to each subgroup to maximize therapeutic effects. However, there is a lack of LPA research that has investigated the relationships between emotion dysregulation and trauma-related mental disorders within a transdiagnostic framework.

In contrast to LPA, network analysis is focused on elucidating the interrelationships among psychopathological symptoms (Isvoranu et al., 2021), and the interconnectedness between risk/protective factors and psychopathological symptoms (Isvoranu et al., 2017; Lunansky et al., 2024). Recent network studies have elucidated the interrelationships between dysregulated negative emotion processes and PTSD (Dieujuste et al., 2025; Haws et al., 2022), as well as dysregulated positive emotion processes and PTSD (Wang & Contractor, 2025; Weiss et al., 2020). Overall, findings from these network studies have identified core emotion dysregulation dimensions with the highest bridge associations with PTSD, and targeting these specific emotion dysregulation dimensions could potentially reduce the co-occurrence of specific PTSD symptoms (Dieujuste et al., 2025; Haws et al., 2022; Wang & Contractor, 2025; Weiss et al., 2020). Limitations of previous network analysis research include the predominant focus on non-clinical and Western samples, lack of transdiagnostic psychopathology network analysis, and predominant use of self-report measures of PTSD (Haws et al., 2022; Weiss et al., 2020).

Although LPA and network analysis address different research questions, recent studies have used both methods in a complementary manner to inform personalized assessment and intervention for physical and mental health conditions (Djelantik et al., 2020; Wang et al., 2024; Xue et al., 2024; Zhang et al., 2025). LPA is first used to identify subgroups of patients and network analysis is subsequently conducted to elucidate the co-occurrence between core symptoms within each subgroup (Wang et al., 2024; Zhang et al., 2025). By elucidating the interrelationships between emotion dysregulation and psychopathology in trauma-exposed individuals, network analysis helps identify core emotion dysregulation processes that maintain or exacerbate PTSD and trauma-related psychiatric comorbidities. Interventions that target core emotion dysregulation processes could disrupt maladaptive feedback loops and therapeutic benefits may spread to other parts of the emotion dysregulation and psychopathology network. This in turn could lead to improvements in overall functioning and well-being. From a translational perspective, findings from this integrated analysis have significant clinical potential to augment treatment targeting, where different groups of patients receive tailored interventions according to their risk profile, while precision medicine-driven interventions target core emotion dysregulation processes.

Building on previous research, the present study investigates the latent profiles and interrelationships between emotion dysregulation and psychopathology (PTSD, dissociation, psychosis, and affective symptoms) in a sample of trauma-exposed patients seeking outpatient treatment for severe mental disorders. The specific aims were to: (1) identify latent profiles of emotion dysregulation (broad ability deficits and habitual use of worry, rumination, and suppression strategies) and psychopathology (PTSD, dissociation, psychosis, and depressive symptoms), and their associations with socio-demographic and clinical variables; (2) determine the interrelationships between emotion dysregulation and psychopathology within each latent profile and the whole sample through network analysis.

## Methods

### Participants

Participants were recruited from the outpatient clinics of the Institute of Mental Health, the sole tertiary psychiatric hospital in Singapore from 2022 to 2024. All patients were clinically assessed by their attending psychiatrist using the Structured Clinical Interview for DSM-IV Clinician Version (SCID-IV-CV; First et al., 1996) to establish a diagnosis of mood disorder or schizophrenia spectrum disorder. The attending psychiatrist also assessed each patient’s capacity to provide informed consent and ability to participate in the present study. Only patients deemed stable and well enough were referred to the research team for consent taking. The research team explained the study procedures to the participant, their family members, and their assigned case manager. Written informed consent was subsequently obtained from each participant in a private and quiet research assessment room in our hospital clinic. All participants completed the interviews and self-report measures in the same room and a trained research psychologist was present to address any queries. The research psychologist checked the responses to the self-report measures and unclear responses were clarified with each participant before they were debriefed, compensated, and thanked for their participation.

The inclusion criteria for this study were: (1) primary diagnosis of mood disorder (depression or bipolar disorder) or schizophrenia spectrum disorder; (2) age between 21 and 65 years; and (3) able to speak and understand English. Participants were excluded if they had severe physical or mental conditions (e.g., intellectual disability). The authors assert that all procedures contributing to this work comply with the ethical standards of the relevant national and institutional committees on human experimentation and with the Helsinki Declaration of 1975, as revised in 2008. All procedures involving human subjects/patients were approved by our hospital’s Institutional Review Board and the Domain Specific Review Board (NHG DSRB Ref: 2022/00759).

### Measures

The Stressful Life Events Screening Questionnaire (SLESQ; Goodman et al., 1998) was used to assess for traumatic or stressful life events. The SLESQ lists 12 potential stressful life events, including a life-threatening illness or accident, robbery or armed assault, physical, emotional or sexual abuse, and witnessing a sudden death of a loved one. A final open-ended question was used to capture other events not listed in the questionnaire. Participants were asked to provide relevant details of each event (i.e., age/month of exposure, frequency, severity, and context). We modified the SLESQ to require participants to identify their index event for subsequent PTSD assessment (Liu et al., 2021, 2024a). In this study, we were interested in assessing PTSD symptoms after exposure to both Criterion A and non-Criterion A events. Hence, participants who endorsed at least one event were subsequently assessed for PTSD symptoms. If any participant endorsed more than one event, they were asked to indicate the most distressing event as the index event. All endorsed events on the SLESQ should have occurred at least one month prior to the PTSD assessment.

The Clinician-Administered PTSD Scale for DSM-5 (CAPS-5; Weathers et al., 2018) was used to assess the severity of PTSD symptoms and trauma-related dissociation. The CAPS-5 assessment was anchored to the index event identified in the SLESQ. In this study, we calculated scores for four PTSD symptom clusters (intrusion, avoidance, negative alterations in cognitions and mood, alterations in arousal and reactivity) and trauma-related dissociation. The CAPS-5 had adequate internal consistency in the present sample (α = 0.89). Trained research psychologists administered the CAPS-5 and adequate inter-rater scores were achieved among the team (Intraclass Correlation Coefficient [ICC] = 0.95).

The Brief Psychiatric Rating Scale-Expanded (BPRS-E; Lukoff et al., 1986) was used to assess for current positive psychotic symptoms, negative psychotic symptoms, and depressive symptoms. The present study calculated composite scores for positive psychotic symptoms (items: hallucinatory behaviour, unusual thought content, conceptual disorganization, and grandiosity), negative psychotic symptoms (items: blunted affect, emotional withdrawal, and motor retardation), and affective symptoms (items: depressive mood, anxiety, guilt feelings, and somatic concern) (Hochstrasser et al., 2022). The BPRS-E had acceptable internal consistency in the present sample (α = 0.74). Trained research psychologists administered the BPRS-E and achieved adequate inter-rater scores among the team (ICC = 0.94).

The Difficulties with Emotion Regulation Scale Short (DERS-S; Kaufman et al., 2016) was used to assess for emotion regulation ability deficits as outlined in the ability-based model of emotion dysregulation. In this study, we calculated composite scores for six dimensions: limited access to adaptive emotion regulation strategies (Poor Strategies), non-acceptance of emotional responses (Non-Acceptance), poor emotional awareness (Poor Awareness), poor emotional clarity (Poor Clarity), difficulties engaging in goal-directed behaviour (Poor Goals), and inability to control impulsive behaviours (Impulsivity) (Kaufman et al., 2016). The DERS had adequate internal consistency in the present sample (α = 0.90).

Worry, rumination, and expressive suppression were conceptualized as maladaptive emotion regulation strategies as outlined in the strategy-based model of emotion dysregulation (Aldao et al., 2010). The Penn State Worry Questionnaire-Abbreviated (PSWQ-A; Hopko at al., 2003) was used to assess for habitual worry. The Ruminative Response Scale short-form (RRS; Treynor et al., 2003) was used to assess for habitual rumination. The subscale of the Emotion Regulation Questionnaire (ERQ-S; Gross & John, 2003) was used to assess for expressive suppression. All three measures had adequate internal consistencies for worry (α = 0.94), rumination (α = 0.84), and expressive suppression (α = 0.77).

The following socio-demographic information was obtained through a self-report questionnaire: age, gender, ethnicity, marital status, education status, and employment status. Psychiatric comorbidities, chronic physical comorbidities, and family history of mental illness were extracted from the participants’ medical records.

### Statistical analyses

LPA was conducted to identify latent profiles of emotion dysregulation and psychopathology (PTSD, dissociation, psychosis, and affective symptoms). Composite scores of maladaptive emotion regulation strategies, emotion regulation ability deficits, PTSD symptom clusters, trauma-related dissociation, positive psychotic symptoms, negative psychotic symptoms, and affective symptoms were rescaled (range 0 to 100) for ease of visual interpretation and included in the model. Model fit indices were evaluated to identify the optimal profile solution: (1) Bayesian information criterion (BIC), (2) Sample-Size Adjusted BIC (SSABIC), (3) Akaike’s information criterion (AIC), (4) log-likelihood, (5) boot-strapped likelihood ratio test (BLRT), (6) Vuong-Lo-Mendell-Rubin test (VLMR), and (7) entropy values (range 0 to 1) (Wang & Wang, 2020). Model selection was based on overall improvement of fit indices, as well as parsimony and interpretability of the model (Wang & Wang, 2020). Further, the proportion of individuals within each latent profile should be >5 % of the total sample (Ferguson et al., 2020). Multinomial logistic regression analysis was conducted to determine the associations between socio-demographic/clinical variables and latent profiles. LPA and multinomial logistic regression analysis were conducted in MPlus (version 8.3) with alpha set at 0.05.

Network analysis was conducted in R (version 4.5.2) using the *bootnet* package (version 1.6) to determine the interrelationships between emotion dysregulation and psychopathology (PTSD, dissociation, psychosis, and affective symptoms). We estimated regularized partial correlation networks and implemented the graphical least absolute shrinkage and selection operator (LASSO) with Extended Bayesian Information Criterion (EBIC) to minimize spurious edges, which is aligned with guidelines in network psychometrics (Isvoranu & Epskamp, 2023). The same LPA indicators were used as nodes in the network analysis. The nodes were further grouped into two communities: the emotion dysregulation community and the psychopathology community.

We calculated two centrality indices to describe the impact of each node within the network: 1) node/bridge strength and 2) node predictability. Node strength and bridge strength refers to the number and strength of connections between nodes or communities respectively. Node predictability refers to how well a node could be predicted by other nodes. We used the non-parametric bootstrap (5000 networks) and 95 % bootstrapped confidence intervals to examine the accuracy of edges in the network (Epskamp et al., 2018). We used the case-dropping bootstrap to compute the correlation stability (CS) coefficient and evaluate the stability of node strength/bridge strength centrality (CS-coefficient ≥ 0.50).

Monte Carlo simulations were conducted to determine the sample size required to detect one to five latent profiles based on 17 indicators. The results revealed that a sample size of N = 450 to 500 provided adequate power (> 80 %) to detect a three-profile solution if the profiles were at least moderately separated (entropy ≥ .84). A sample size of at least 500 is also recommended for network analysis (Epskamp & Fried, 2018; Nehler & Schultze, 2024).

## Results

### Sample characteristics

The study team were referred a total of N = 596 patients as potential participants. A total of N = 500 outpatients with mood disorders and schizophrenia spectrum disorders (M*_age_* = 35.47 years, Female sex = 58.8 %) provided informed consent and agreed to participate in this study (response rate = 83.8 %). Overall, 92.2 % (N = 461) of the sample were exposed to at least one traumatic or stressful life event. Table 1 summarizes the socio-demographic and clinical characteristics of this sample. Table 2 presents the correlation table for all indicators or nodes in the LPA and network analysis respectively.

**Table 1:** Socio-demographics and clinical characteristics.

Variable (N = 461)	N (%) / M ± SD
Age	35.47 ± 10.91
Gender	
Male	190 (41.2)
Female	271 (58.8)
Ethnicity	
Chinese	316 (68.5)
Malay	79 (17.1)
Indian	37 (8.0)
Others	29 (6.3)
Marital Status	
Single	397 (86.1)
Married	64 (13.9)
Education	
Primary and below	14 (3.0)
Secondary	163 (35.4)
Pre-Tertiary	162 (35.1)
Tertiary	122 (26.5)
Employment	
Employed	302 (65.5)
Unemployed	159 (34.5)
Stressful Life Events	
Life threatening physical illness	218 (47.1)
Emotional abuse	246 (53.1)
Traumatic psychosis	62 (13.4)
Bullying	20 (4.3)
Traumatic Events	
Life threatening accident	70 (15.1)
Armed robbery	20 (4.3)
Sudden death of family member or close friend	121 (26.1)
Sexual assault	80 (17.3)
Molestation	146 (31.5)
Childhood physical abuse	217 (46.9)
Physical assault	114 (24.6)
Threatened with deadly weapon	84 (18.1)
Witnessed physical or sexual assault	58 (12.5)
Attempted suicide or witnessed suicide	10 (2.2)
Primary Psychiatric Diagnosis	
Mood disorder	237 (51.4)
Schizophrenia spectrum disorder	224 (48.6)
Cumulative Psychiatric Comorbidity	0.52 ± 0.75
≥ 2 comorbid conditions	44 (11.6)
1 comorbid condition	140 (30.4)
None	277 (60.1)
Chronic Physical Comorbidity	
Yes	308 (66.8)
No	153 (33.2)
Family History of Mental Illness	
Mood disorders	32 (6.9)
Schizophrenia spectrum disorders	28 (6.1)
Others (anxiety disorders, substance use disorder, suicide)	49 (10.6)
Multiple conditions	32 (6.9)
None	320 (69.4)

Note: Psychiatric Comorbidity = Depression, eating disorders, generalized anxiety disorder, obsessive compulsive disorder, personality disorders, post-traumatic stress disorder, social anxiety disorder.

Chronic Physical Comorbidity = Arthritis, arthropathy, asthma, chronic eczema, chronic ischemic heart disease, diabetes, gastritis, HIV infection, hypertension, hyperlipidemia, hypothyroidism, irritable bowel syndrome, kidney failure, migraine, menstrual disorder, neurological conditions, scoliosis.

**Table 2:** Correlations among emotion dysregulation and trauma-related psychopathology variables.

	DERS1	DERS2	DERS3	DERS4	DERS5	DERS6	PSWQ	RRS	ERQ	CAPS1	CAPS2	CAPS3	CAPS4	CAPS5	BPRS1	BPRS2	BPRS3
DERS1	1																
DERS2	.59***	1															
DERS3	.59***	.62***	1														
DERS4	-.05	-.02	-.06	1													
DERS5	.70***	.71***	.66***	-.001	1												
DERS6	.54***	.44***	.46***	.15***	.56***	1											
PSWQ	.58***	.53***	.49***	-.06	.60***	.43***	1										
RRS	.59***	.50***	.44***	-.18***	.55***	.39***	.58***	1									
ERQ	.31***	.19***	.19***	.15****	.35***	.34***	.31***	.31***	1								
CAPS1	.42***	.38***	.32***	.05	.39***	.38***	.33***	.41***	.22***	1							
CAPS2	.40***	.33***	.28***	.09	.37***	.36***	.29***	.34***	.23***	.64***	1						
CAPS3	.53***	.48***	.36***	.10*	.60***	.51***	.48***	.49***	.35***	.60***	.53***	1					
CAPS4	.49***	.43***	.37***	.07	.48***	.45***	.38***	.42***	.27***	.57***	.53***	.66***	1				
CAPS5	.38***	.32***	.22***	.06	.39***	.37***	.27***	.38***	.21***	.49***	.37***	.54***	.51***	1			
BPRS1	.13**	.13**	.22***	.01	.18***	.24***	.13**	.17***	.15***	.16***	.13**	.19***	.18***	.31***	1		
BPRS2	.05	.08	.07	.16***	.12**	.07	.03	.01	.20***	.13**	.12**	.21***	.11*	.11*	.07	1	
BPRS3	.58***	.48***	.41***	-.02***	.58	.45***	.58***	.51***	.27***	.56***	.45***	.68***	.60***	.44***	.20***	.04	1

Note: DERS1 = Non-acceptance of emotions; DERS2 = Difficulties engaging in goal-directed behaviour; DERS3 = Impulsivity; DERS4 = Poor emotional awareness; DERS5 = Limited access to adaptive emotion regulation strategies; DERS6 = Poor emotional clarity; PSWQ = Worry; RRS = Rumination; ERQ = Expressive suppression; CAPS1 = Intrusions; CAPS2 = Avoidance; CAPS3 = Alterations in cognition and mood; CAPS4 = Alterations in arousal and reactivity; CAPS5 = Trauma-related dissociation; BPRS1 = Positive symptoms; BPRS2 = Negative symptoms; BPRS3 = Affective symptoms. **p < 0.01; ***p <.001.

### Latent profiles of emotion dysregulation and trauma-related psychopathology

**Fig. 1:**
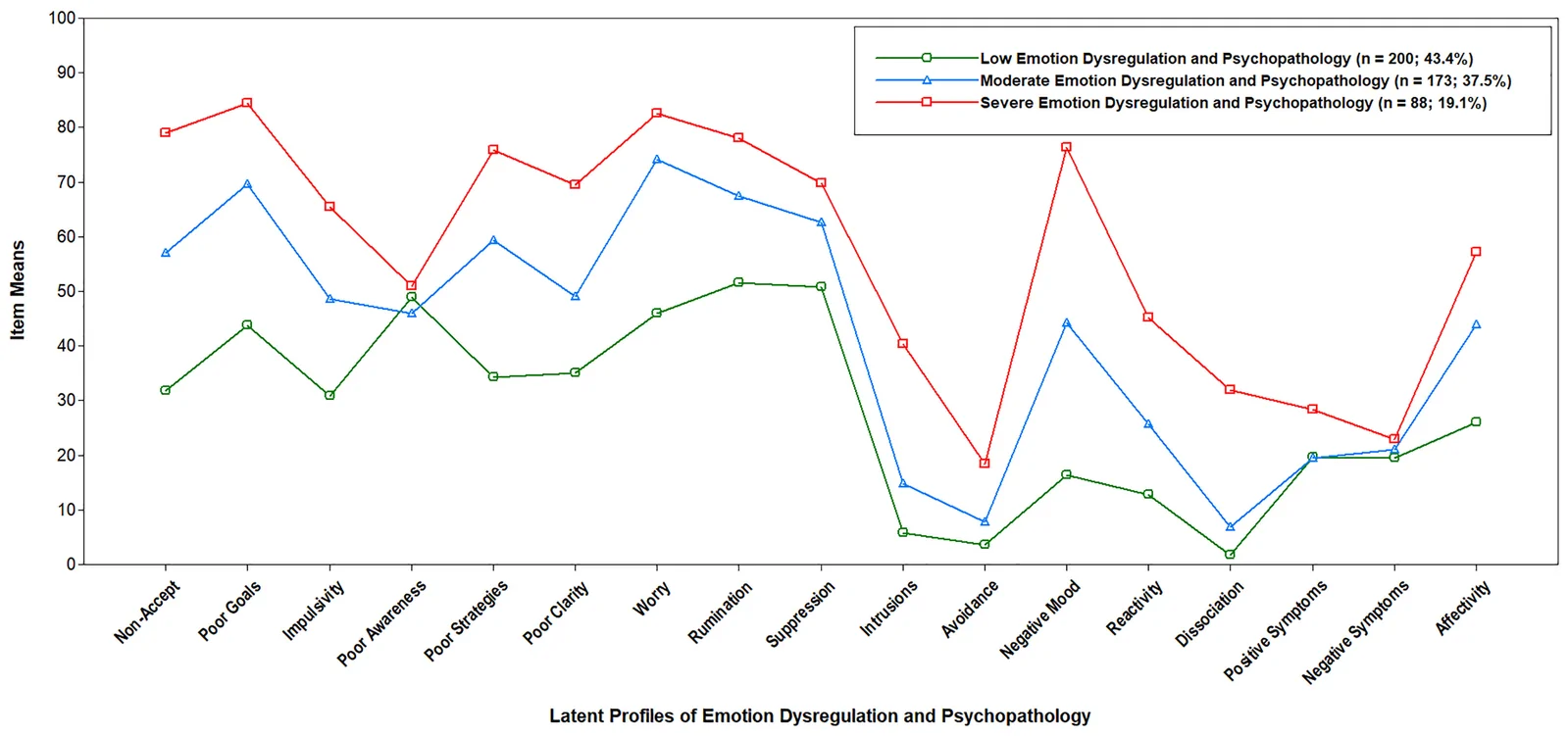
Latent Profiles of Emotion Dysregulation and Trauma-related Psychopathology.

A series of models ranging from 1 to 5 latent profiles were specified and estimated. Table 3 summarizes the fit indices for each model. While the BIC, SSABIC, and AIC decreased from one-profile to five-profile solutions, the VLMR test was not significant for the four-profile and five-profile solutions. Hence, the three-profile solution was deemed most optimal and the latent profile plot is shown in Fig. 1. Profile 1 comprised individuals with the lowest levels of emotion dysregulation and psychopathology; Profile 1 was labelled “Low emotion dysregulation and psychopathology” (*n* = 200; 43.4 %). Profile 2 comprised individuals with higher levels of emotion dysregulation and psychopathology than Profile 1. Profile 2 was labelled “Moderate emotion dysregulation and psychopathology” (*n* = 173; 37.5 %). Profile 3 comprised individuals with the highest levels of emotion dysregulation and psychopathology. Hence, Profile 3 was labelled “Severe emotion dysregulation and psychopathology” (*n* = 88; 19.1 %).

**Table 3:** Fit indices for latent profile models 1–5.

Model	BIC	SSABIC	AIC	Log likelihood	BLRT	VLMR	Entropy	Profile Proportions
1	67,918.14	67,810.23	67,777.61	−33,854.80	-	-	-	-
2	65,752.51	65,587.47	65,537.57	−32,716.78	*p* < .001	*p* < .001	0.92	Profile 1 = 272 (59%) Profile 2 = 189 (41%)
**3**	**65,185.46**	**64,963.30**	**64,896.12**	**−32,378.06**	***p* < .001**	***p* = 0.01**	**0.91**	**Profile 1 = 200 (43%)** **Profile 2 = 173 (38%)** **Profile 3 = 88 (19%)**
4	65,030.59	64,751.30	64,666.85	−32,245.42	*p* < .001	*p* = 0.23	0.90	Profile 1 = 165 (36%) Profile 2 = 163 (35%) Profile 3 = 70 (15%) Profile 4 = 63 (14%)
5	64,922.54	64,586.13	64,484.40	−32,136.20	*p* < .002	*p* = 0.36	0.93	Profile 1 = 173 (38%) Profile 2 = 141 (31%) Profile 3 = 62 (13%) Profile 4 = 43 (9%) Profile 5 = 42 (9%)

Note: AIC = Akaike information criterion; BIC = Bayesian information criterion; SSABIC = sample size adjusted BIC; BLRT = Boot-strapped likelihood ratio test; VLMR = Vuong-Lo-Mendell-Rubin test. The optimal model is highlighted in bold.

### Associations between latent profiles and socio-demographic and clinical variables

Younger age, any chronic physical comorbidities (binary), and cumulative psychiatric comorbidities were significantly associated with Profile 3 and Profile 2. Additionally, Malay ethnicity and unemployment status were significantly associated with Profile 3. The multinomial logistic regression results are presented in Table 4.

**Table 4:** Multinomial logistic regression.

	Profile 2 vs. Profile 1			Profile 3 vs. Profile 1		
	Odds Ratio	95% CI	*p*-value	Odds Ratio	95% CI	p-value
Age	0.95*	0.92 to 0.97	< .001	0.91*	0.88 to 0.94	< .001
Gender						
Male	0.90	0.58 to 1.40	0.66	0.73	0.41 to 1.29	0.28
Female	Ref.			Ref.		
Ethnicity						
Malay	1.44	0.74 to 2.77	0.27	4.64*	2.27 to 9.46	< .001
Indian	1.14	0.51 to 2.56	0.73	1.59	0.56 to 4.47	0.37
Others	1.24	0.49 to 3.16	0.64	2.55	0.88 to 7.37	0.08
Chinese	Ref.			Ref.		
Marital Status						
Single	0.76	0.40 to 1.44	0.40	0.96	0.38 to 2.39	0.93
Married	Ref.			Ref.		
Education Status						
Primary and below	1.17	0.29 to 4.64	0.82	1.38	0.29 to 6.45	0.67
Secondary and above	Ref.			Ref.		
Employment Status						
Unemployed	1.18	0.73 to 1.90	0.48	1.86*	1.03 to 3.37	0.03
Employed	Ref.			Ref.		
Cumulative Psychiatric Comorbidity	1.51*	1.11 to 2.06	0.01	1.47*	1.01 to 2.15	0.04
Family History of Mental Illness						
Yes	0.80	0.50 to 1.29	0.37	0.91	0.50 to 1.66	0.77
No	Ref.			Ref.		
Chronic Physical Comorbidity						
Yes	1.64*	1.02 to 2.62	0.03	2.08*	1.12 to 3.86	0.01
No	Ref.			Ref.		

Note: Significant Odds Ratios (*p* < 0.05) were denoted with *.

### Emotion dysregulation and psychopathology network of Profile 1

**Fig. 2:**
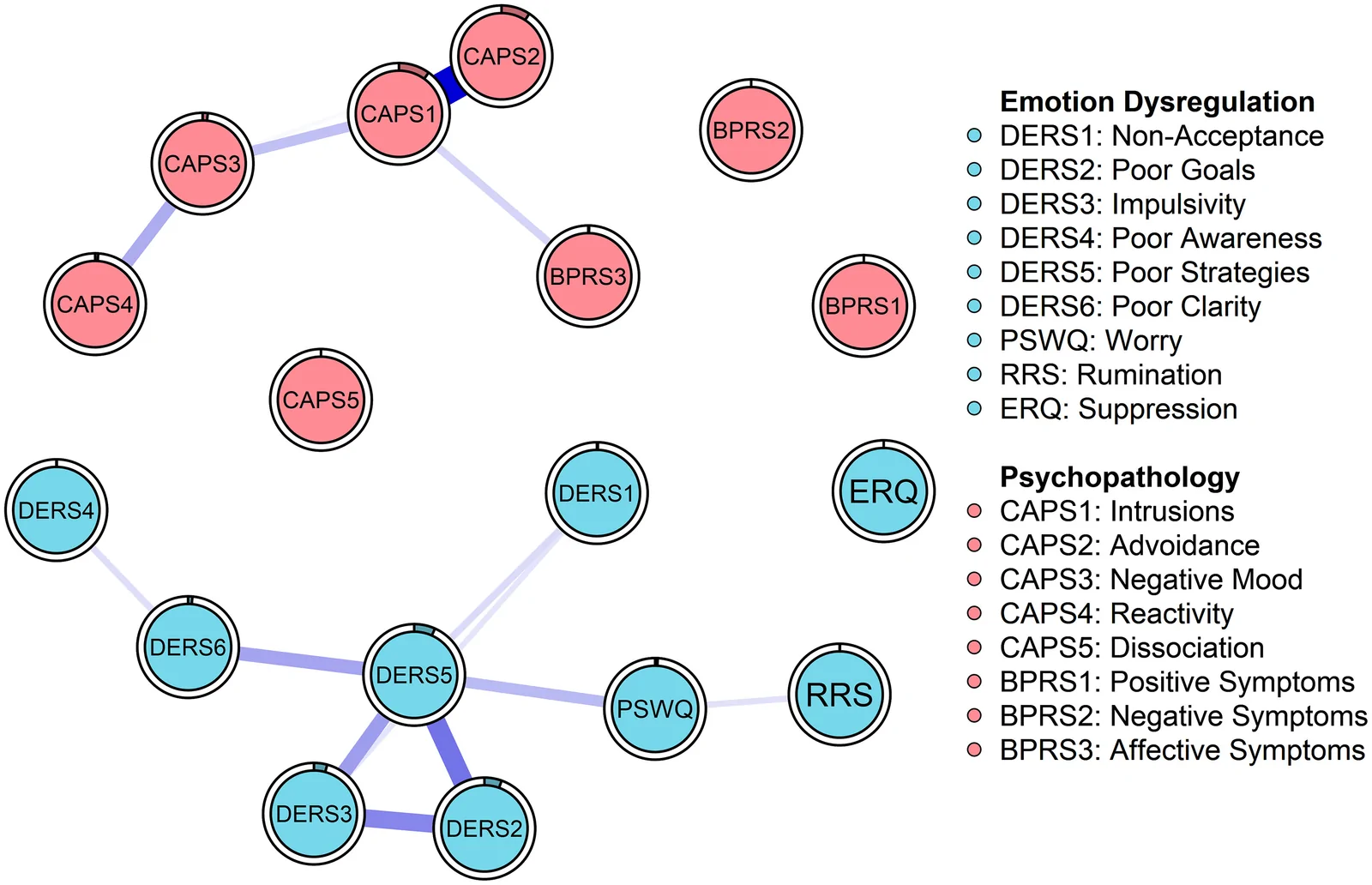
Emotion dysregulation and trauma-related psychopathology network for Profile 1 (N = 200). Blue edge = positive association; Thicker and darker edges indicate stronger associations; Rings around nodes convey predictability, with shadowed parts depicting variance explained by connected nodes.

The emotion dysregulation and psychopathology network for Profile 1 is presented in Fig. 2. The strongest nodes in the network were PTSD symptoms of intrusion (CAPS1) for the psychopathology community and limited access to adaptive emotion regulation strategies (DERS5) for the emotion dysregulation community (Supplementary Figure 1). There were intra-group connections for the emotion dysregulation community, where limited access to adaptive emotion regulation strategies (DERS5) was positively associated with other emotion dysregulation dimensions, such as the inability to control impulsive behaviours (DERS3) and worry (PSWQ). There were also intra-group connections for the psychopathology community, where PTSD symptoms of intrusion (CAPS1) were positively associated with affective symptoms (BPRS3) and other PTSD symptoms. While intra-group connections were present, there were no inter-group connections between emotion dysregulation and psychopathology communities.

### Emotion dysregulation and psychopathology network of Profile 2

**Fig. 3:**
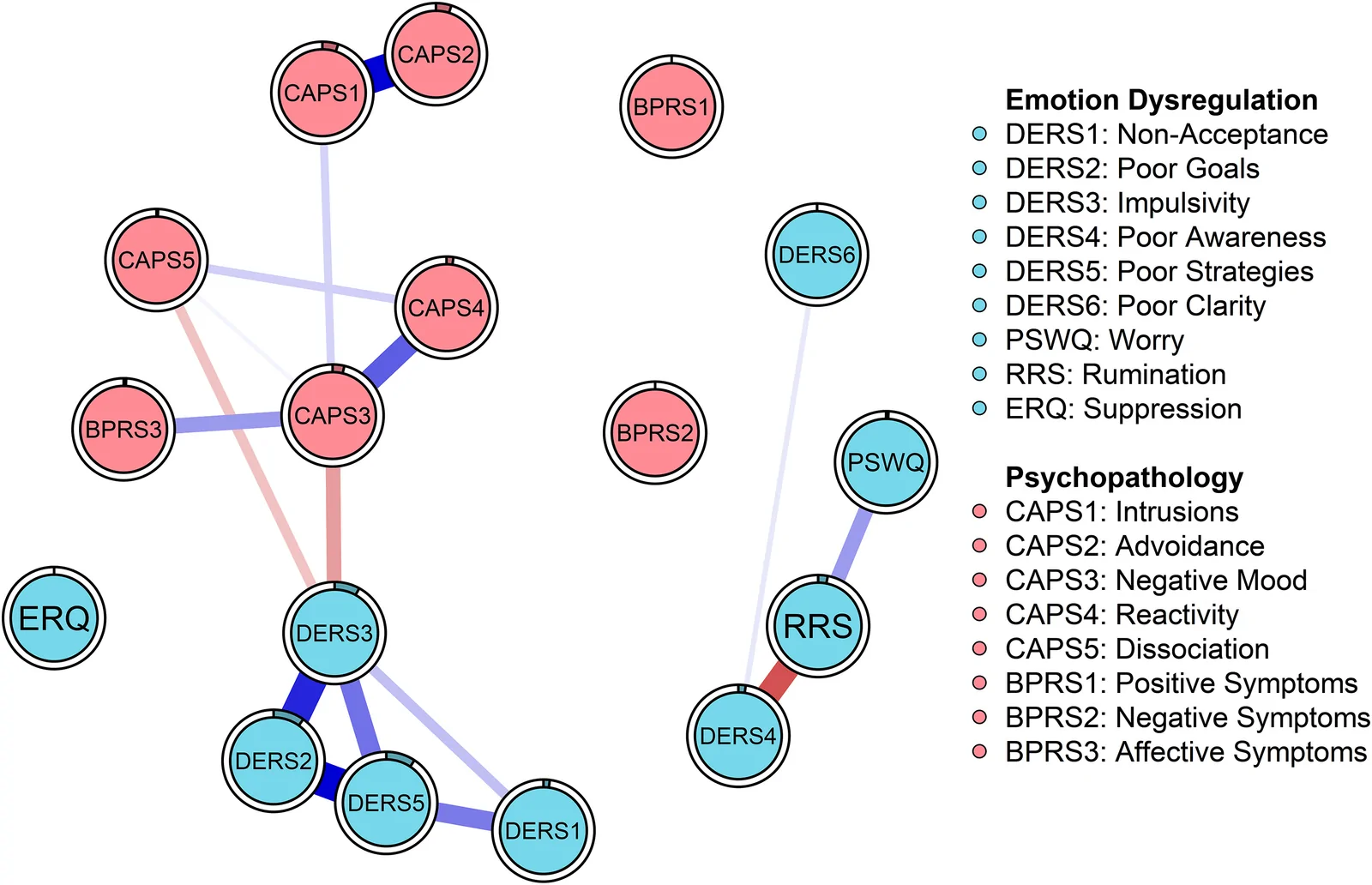
Emotion dysregulation and trauma-related psychopathology network for Profile 2 (N = 173). Blue edge = positive association; Red edge = negative association; Thicker and darker edges indicate stronger associations; Rings around nodes convey predictability, with shadowed parts depicting variance explained by connected nodes.

The emotion dysregulation and psychopathology network for Profile 2 is presented in Fig. 3. The strongest nodes in the network were PTSD symptoms of negative alterations in cognitions and mood (CAPS3) for the psychopathology community and inability to control impulsive behaviours (DERS3) for the emotion dysregulation community (Supplementary Figure 2). In contrast to Profile 1, Profile 2 had inter-group connections between emotion dysregulation and psychopathology communities. Specifically, the inability to control impulsive behaviours (DERS3) had the highest bridge strength (Supplementary Figure 3). There was a negative association between inability to control impulsive behaviours (DERS3) and PTSD symptoms of negative alterations in cognitions and mood (CAPS3).

### Emotion dysregulation and psychopathology network of Profile 3

**Fig. 4:**
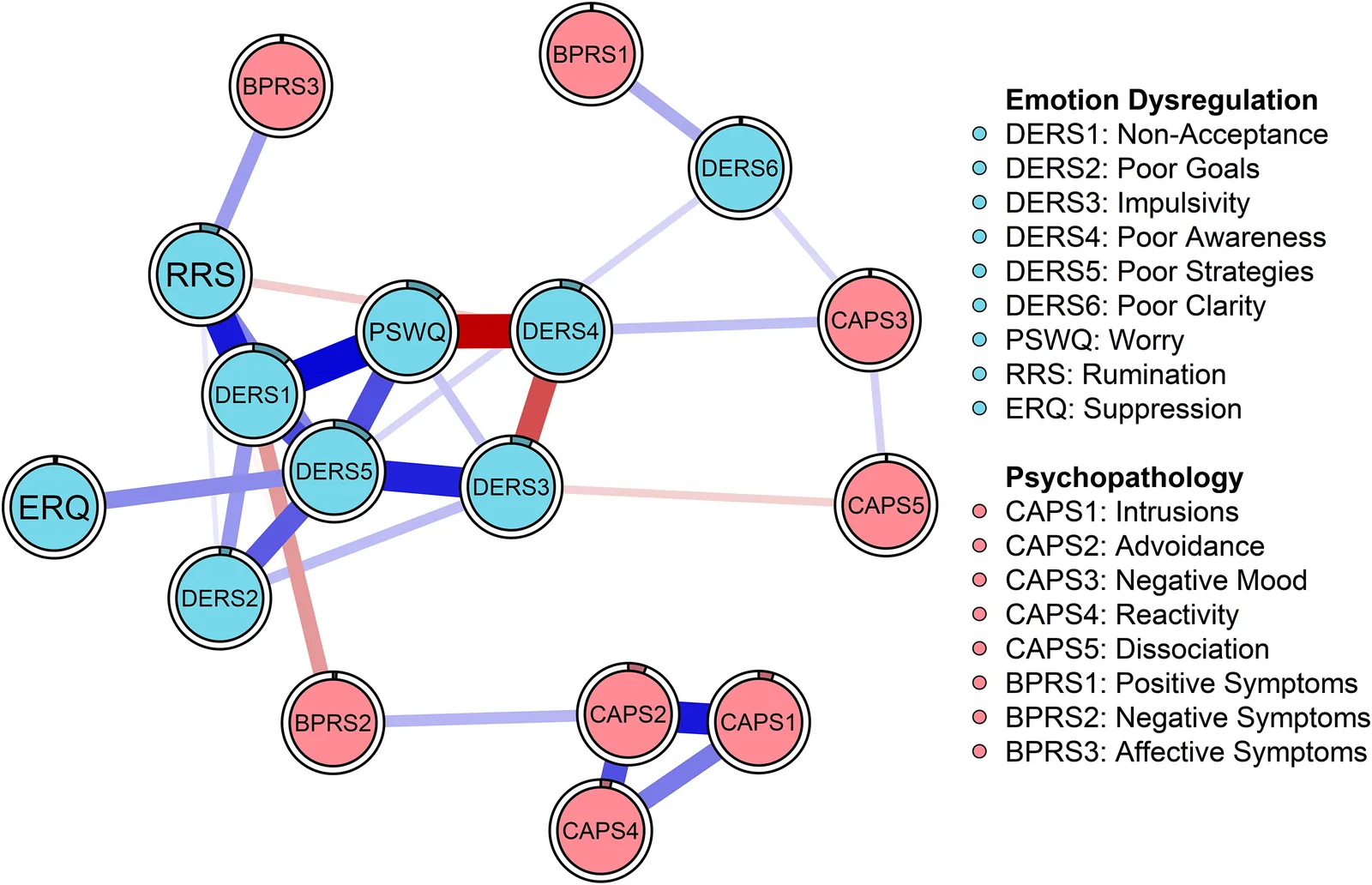
Emotion dysregulation and trauma-related psychopathology network for Profile 3 (N = 88). Blue edge = positive association; Red edge = negative association; Thicker and darker edges indicate stronger associations; Rings around nodes convey predictability, with shadowed parts depicting variance explained by connected nodes.

The emotion dysregulation and psychopathology network for Profile 3 is presented in Fig. 4. Profile 3 had the highest emotion dysregulation network density where multiple emotion dysregulation nodes (i.e., both strategies and abilities) clustered closely together. The strongest nodes in the network were PTSD symptoms of avoidance (CAPS2) for the psychopathology community and limited access to adaptive emotion regulation strategies (DERS5) for the emotion dysregulation community (Supplementary Figure 4). In contrast to Profile 2, Profile 3 had more inter-group connections between emotion dysregulation and psychopathology communities. Poor emotional clarity (DERS6) had the highest bridge strength (Supplementary Figure 5). Poor emotional clarity (DERS 6) was positively associated with positive psychotic symptoms (BPRS 1) and PTSD symptoms of negative alterations in cognitions and mood (CAPS3).

### Emotion dysregulation and psychopathology network of whole sample

**Fig. 5:**
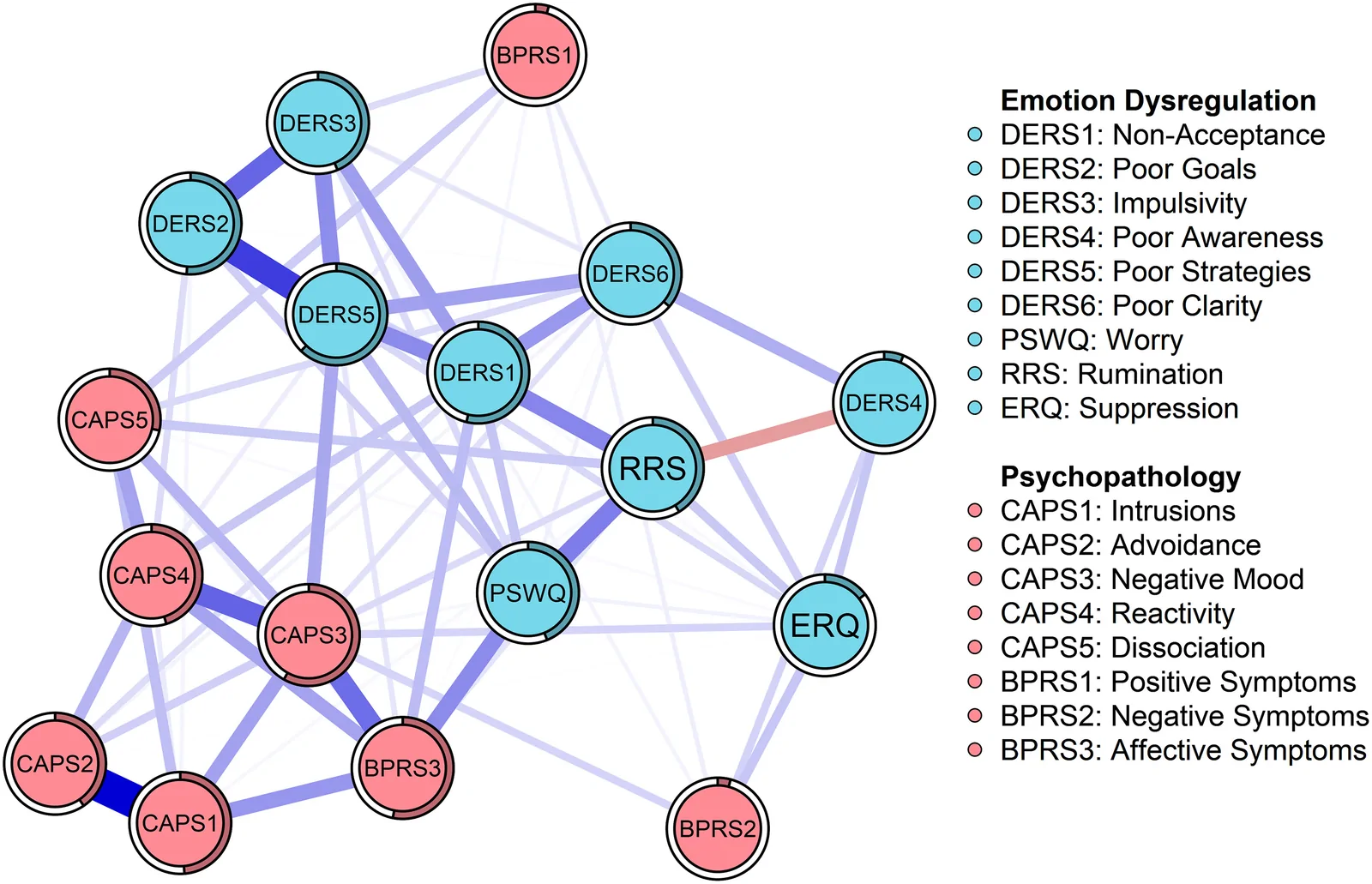
Emotion dysregulation and trauma-related psychopathology network for the whole sample (N = 461). Blue edge = positive association; Red edge = negative association; Thicker and darker edges indicate stronger associations; Rings around nodes convey predictability, with shadowed parts depicting variance explained by connected nodes.

The emotion dysregulation and trauma-related psychopathology network for the whole sample is presented in Fig. 5. The strongest nodes in the network were PTSD symptoms of negative alterations in cognitions and mood (CAPS3) for the psychopathology community and limited access to adaptive emotion regulation strategies (DERS5) for the emotion dysregulation community (Supplementary Figure 6). Affective symptoms (BPRS3) had the highest bridge strength (Supplementary Figure 7). Affective symptoms (BPRS3) were positively associated with worry (PSWQ), non-acceptance of emotional responses (DERS1), and limited access to adaptive emotion regulation strategies (DERS5).

## Discussion

The present study adopted a transdiagnostic approach and synergized the use of LPA (to identify varying risk profiles of emotion dysregulation and psychopathology) and network analysis (to elucidate the interrelationships between emotion dysregulation and psychopathology within each profile). LPA analysis revealed three latent profiles of emotion dysregulation and psychopathology that varied in degree rather than in kind. However, there were important nuances observed. Although all three latent profiles did not differ in mean levels of poor emotional awareness (i.e., whether one pays attention to his/her emotions), there were clear differences in mean levels of poor emotional clarity (i.e., whether one is able to recognize and understand his/her emotions) across three latent profiles. Profile 3 had significantly higher levels of poor emotional clarity than poor emotional awareness, while Profile 1 had significantly higher levels of poor emotional awareness than poor emotional clarity. Previous studies have studied the interplay between poor emotional clarity and poor emotional awareness (Dieujuste et al., 2025; Pugach & Wisco, 2023). A recent study conducted LPA of emotion regulation strategies among undergraduate students and reported that poor emotional clarity, but not poor emotional awareness, was a significant predictor of profile classification (Pugach & Wisco, 2023). Previous research on alexithymia shows that difficulties in recognizing one’s emotions had the strongest association with PTSD (Edwards, 2022). Overall, our results are aligned with current understanding in the field, which suggests that poor emotional awareness and poor emotional clarity are inter-related yet distinct constructs; hence, being aware of one’s emotions does not necessarily mean that one has a clear understanding of one’s emotions (Dieujuste et al., 2025).

In terms of psychopathology, Profile 3 endorsed elevated PTSD symptoms of negative alterations in cognition and mood and affective symptoms compared to other types of psychopathology within the same profile. Further, Profile 3 had elevated levels of trauma-related dissociation and positive psychotic symptoms compared to Profiles 1 and 2. Taken together, these findings suggest that cognitive-affective disturbances and dissociative phenomena are more severe symptoms that occur across the spectrum of severe mental disorders (Hardy, 2017; Isvoranu et al., 2017; Liu et al., 2025). In addition to elevated psychopathology, Profile 3 had the highest levels of emotion dysregulation. This finding highlights that emotion dysregulation could be a critical precipitating and perpetuating risk factor for severe psychopathology. Our finding also supports the transdiagnostic role of emotion dysregulation in understudied psychopathology, such as dissociation and psychosis (Ford, 2025; Liu et al., 2021). This view is supported by the DT framework, where severe emotion dysregulation may be considered a significant individual difference that contributes to symptom heterogeneity and psychiatric comorbidity after complex trauma (Cruz et al., 2022).

There were novel associations between the latent profiles and socio-demographic and clinical correlates. Profiles 2 and 3 were associated with younger age, physical and psychiatric comorbidities, while Profile 3 was additionally associated with Malay ethnicity and unemployment status. With reference to the DT framework and COR theory (Cruz et al., 2022; Hobfoll et al., 2011), cumulative trauma over the lifespan and serious disturbances to childhood socioemotional development are associated with compounding resource losses, which in turn create vulnerabilities for multiple physical and psychiatric comorbidities. Moreover, unemployment status contributes to aggravated resource loss, where vulnerable individuals may experience severe financial strain and major psychosocial challenges (Merrill et al., 2026; Virgolino et al., 2022). Relatedly, unemployment could be perceived as a traumatic event and contributes to both PTSD and complex PTSD in at-risk individuals (Nvo-Fernandez et al., 2025). It is not fully clear as to why Malay ethnicity was significantly associated with Profile 3. A population epidemiological survey reported that Malay ethnicity had higher odds of obsessive compulsive disorder (OCD) compared to Chinese ethnicity (Subramaniam et al., 2020). OCD is associated with trauma and emotion dysregulation in the literature (See et al., 2022; Wislocki et al., 2023); hence, more research is required to elucidate the associations among Malay ethnicity, severe emotion dysregulation, and trauma-related psychopathology. Notably, younger age was strongly associated with Profiles 2 and 3. This finding suggests severe emotion dysregulation and psychopathology are prevalent among youth. Converging research evidence shows that emotion dysregulation and psychopathology co-occur and negatively impact youth mental health (Lynch et al., 2021; Roach et al., 2023).

Network analysis identified distinct emotion dysregulation and psychopathology networks for each latent profile. A key finding is that inter-group connections between emotion dysregulation and psychopathology became more salient in Profiles 2 and 3 than Profile 1. Relatedly, inter-group connections between emotion dysregulation and psychopathology increased linearly in tandem with increasing severity of emotion dysregulation and psychopathology from Profile 2 to Profile 3. Taken together, our findings support current research on elucidating subtypes of mental disorders (e.g., PTSD subtypes) (Chiappini et al., 2025; Tull et al., 2020; Zhang et al., 2025). Using PTSD subtypes as a prime example, the network for Profile 1 supports the classic PTSD as a memory disorder and characterised by hallmark intrusive symptoms. This interpretation is supported by the observation that intrusive symptoms was the strongest psychopathology node and there were no inter-group connections between emotion dysregulation and psychopathology. In contrast to Profile 1, the networks for Profiles 2 and 3 demonstrate inter-group connections between emotion dysregulation and psychopathology, which suggest a more central role of emotion dysregulation in PTSD symptomatology and trauma-related psychiatric comorbidities (e.g., dissociation and psychosis). Hence, more severe PTSD may be conceptualized as a disorder of emotion in addition to a memory disorder (Tull et al., 2020).

Examining the nuances between Profile 2 and Profile 3 revealed several interesting findings. In Profile 2, there was a negative association between inability to control impulsive behaviours (DERS3) and PTSD symptoms of negative alterations in cognitions and mood (CAPS3). This indicates that when one is upset, he/she may have control of his/her behaviours, but this is associated with more PTSD symptoms of negative alterations in cognitions and mood. While this finding may seem contradictory, it could be understood through the lens of Asian culture where suppression of negative emotions and antagonizing behaviours are normalized to preserve social harmony (Qiu et al., 2025). It should be noted, however, that this is speculative and more research is required to replicate this result. Overall, current research evidence shows that suppression of both behaviours and emotions is stressful and habitual use of this coping strategy could contribute to an exacerbation of internalizing symptoms (Qiu et al., 2025).

In contrast to Profile 2, emotion dysregulation had a more significant role in Profile 3 as evident by the highest emotion dysregulation network density where multiple emotion dysregulation nodes (both abilities and strategies) clustered closely together. In our study, poor emotional clarity was identified as key bridging node with psychopathology. Our finding is aligned with a recent network analysis study which similarly identified poor emotional clarity as key bridging node with PTSD symptoms in a different vulnerable sample comprising socioeconomically marginalized, trauma-exposed and primarily Black women at an elevated risk for psychopathology (Dieujuste et al., 2025). From a COR theory perspective (Hobfoll, 2011), emotional clarity could be severely depleted in trauma-exposed populations with multiple aggravating risk factors, which comprises the inability to flexibly engage in a wide range of adaptive emotion regulation strategies and other helpful coping behaviours (Bonanno et al., 2023). Converging empirical evidence supports this view where poor emotional clarity was found to mediate the association between maladaptive emotion regulation strategies and positive psychotic symptoms in schizophrenia spectrum disorders (Liu et al., 2020b).

### Clinical implications

Traditional diagnostic approaches are focused on psychiatric symptoms and do not explicitly and comprehensively assess for emotion dysregulation dimensions. Further, traditional diagnostic approaches are not trauma-informed and may not adequately account for trauma-related psychiatric comorbidities. Hence, the association between specific aspects of emotion dysregulation and trauma-related psychiatric comorbidities is often not clearly established in clinical formulations. Accordingly, clinicians may synergize the use of LPA and network analysis as a personalized transdiagnostic assessment that accounts for emotion dysregulation (as a transdiagnostic risk factor), PTSD, and trauma-related mental disorders in a unified approach. LPA is able to identify distinct patient profiles and network analysis of each profile provides information on the co-occurrence between emotion dysregulation and psychopathology to inform personalized interventions. Clinicians may use this information to select and tailor interventions for PTSD and trauma-related psychiatric comorbidity. For example, the network for Profile 1 identified intrusive symptoms as a key node and there were no inter-group connections between emotion dysregulation and psychopathology. Given that PTSD appears to be more characteristic of a memory disorder in Profile 1, gold-standard trauma-focused cognitive behavioural therapies (CBT), such as prolonged exposure and cognitive processing therapy could be used to effectively treat intrusive symptoms (Iyadurai et al., 2019; McLean et al., 2024; Resick et al., 2024). Although gold-standard trauma-focused CBT do not explicitly target emotion dysregulation, core techniques such as imaginal exposure to distressing thoughts and negative emotions may contribute to improved emotion regulation abilities (e.g., emotional acceptance) and reduction in maladaptive emotion regulation strategies (Tull et al., 2020). The indirect positive influence on emotion regulation may have protective downstream effects to prevent the exacerbation of trauma-related psychopathological symptoms for individuals in Profile 1.

The networks for Profiles 2 and 3 show more inter-group connections between emotion dysregulation and psychopathology; hence, individuals in Profiles 2 and 3 would benefit from emotion regulation interventions, such as STAIR and the UP. However, the emotion regulation interventions would be applied differently between Profiles 2 and 3. For individuals in Profile 2, emotion regulation interventions could be tailored to support healthy and culturally-sensitive expression of behaviours and emotions instead of engaging in behavioural suppression. Specifically, clinicians should focus more on behavioural activation than improving emotional clarity for individuals in Profile 2. On the other hand, clinicians should focus more on improving emotional clarity for individuals in Profile 3. This would involve devoting more sessions to emotion labelling and differentiation. Current manuals of emotion regulation interventions (e.g., STAIR) often have one to two sessions dedicated to improving emotional clarity and emotional awareness (Cloitre et al., 2020). Given the severity of emotion dysregulation in Profile 3, at least half of the sessions should focus on building skills capacity in emotional clarity and emotional awareness before moving to other skills. Subsequent sessions should prioritize drawing clear links between emotional clarity/awareness with other concepts of emotion regulation to reinforce learnt skills. In view of the strong associations among emotion dysregulation, PTSD, and trauma-related psychopathology in Profile 3, phase-based trauma interventions are recommended where emotion regulation skills are developed in the first phase to prepare the client for trauma processing in the second phase (Cloitre et al., 2020; Corrigan et al., 2020). The phase-based approach is effective for clients who are in severe distress, unable to tolerate immediate trauma processing, and at-risk of dropping out of treatment (Cloitre et al., 2020; Corrigan et al., 2020; Hundt et al., 2020). Moreover, addressing emotion regulation difficulties in the first phase supports a transdiagnostic approach to effectively treat trauma-related psychiatric comorbidities (e.g., severe dissociation and psychosis) that may contraindicate subsequent PTSD treatment (van Minnen et al., 2012).

### Strengths and limitations

A key strength of the present study is the translational value of synergizing LPA and network analysis. While traditional regression and factor analyses may be helpful to examine the associations between emotion dysregulation and psychopathology and identify underlying latent factors respectively, these investigations neither identify subgroups of patients nor elucidate the co-occurrence between emotion dysregulation dimensions and multiple psychiatric symptoms. Moreover, the results from traditional regression and factor analyses are aggregated and not personalized to subgroups of patients. This significantly limits the translation of research findings to personalized assessment and intervention. Hence, the synergistic use of LPA and network analysis offers vital translational insights above and beyond traditional statistical approaches and this information will advance the field of personalized assessment and intervention. Additional strengths of this study include recruiting a large clinical sample and using interviewer-rated measures to assess for trauma-related psychopathology (e.g., gold standard CAPS-5 for PTSD).

The present study has some limitations that must be considered. First, our study used a cross-sectional design and we were not able to determine causality or directionality of the association between emotion dysregulation and trauma-related psychopathology. Future longitudinal research is required to examine the stability of the latent profiles and network structures over time. Second, future research should include an independent sample to determine the replicability of the latent profiles and network structures. Third, the present study had a high response rate (83.8 %) and this could have introduced some selection bias. Finally, our study was conducted in a clinical sample and our findings may not be generalizable to healthy populations and community samples. Further, our analyses were focused on a transdiagnostic severe mental disorder sample and our results may not generalize to specific diagnostic subgroups. Accordingly, supplementary analyses were conducted to determine if the latent profiles or network structures differed by diagnostic subgroup (mood disorders versus schizophrenia spectrum disorders). Our results revealed three similar latent profiles and network structures for both diagnostic subgroups that closely mirrored the main findings. However, there were more patients with mood disorders (73.3 %) than patients with schizophrenia spectrum disorders (35.7 %) in the latent Profiles 2 and 3. Our results are aligned with the extant literature where emotion dysregulation and affective symptoms occur in both diagnostic subgroups, but are more severe in patients with mood disorders (Anticevic et al., 2015; De Prisco et al., 2023; Liu et al., 2024b, 2020c).

### Conclusion

This is the first transdiagnostic study to deepen our understanding on the co-occurrence between emotion dysregulation and trauma-related psychopathology in a large clinical sample exposed to trauma and stressful life events. Through integrating both LPA and network analysis, we have demonstrated the synergy of both approaches and provided the blueprint to translate person-specific latent profiles and network models into personalized assessments and interventions.

## Funding

This work was supported by the National Medical Research Council (NMRC) Open-Fund Young Investigator Grant (OF-YIRG) (grant number, MOH-OFYIRG21nov-0020).

## Declaration of competing interest

None.

## Appendix Supplementary materials

Image, application 1

Image, image 2 dummy alt text

Image, image 3 dummy alt text

Image, image 4 dummy alt text

Image, image 5 dummy alt text

Image, image 6 dummy alt text

Image, image 7 dummy alt text

Image, image 8 dummy alt text

## References

[bib0001] Anticevic A., Schleifer C., Cho T. (2015). Emotional and cognitive dysregulation in schizophrenia and depression: Understanding common and distinct behavioral and neural mechanisms. Dialogues in Clinical Neuroscience.

[bib0002] Aldao A., Nolen-Hoeksema S., Schweizer S. (2010). Emotion-regulation strategies across psychopathology: A meta-analytic review. Clinical Psychology Review.

[bib0003] American Psychiatric Association (2022).

[bib0004] Barlow D.H. (2018).

[bib0005] Bonanno G.A., Chen S., Galatzer-Levy I.R. (2023). Resilience to potential trauma and adversity through regulatory flexibility. Nature Reviews Psychology.

[bib0006] Chen M.S., Li S.M., Bonanno G.A. (2026). Emotion regulation flexibility and momentary psychopathology symptoms among trauma-exposed veterans with and without posttraumatic stress disorder. Journal of Traumatic Stress.

[bib0007] Chiappini S., Sampogna G., Ventriglio A., Menculini G., Ricci V., Pettorruso M., Martinotti G. (2025). Exploring emerging psychopathological characteristics and challenges of novel depression subtypes: Insights from the literature. Frontiers in Psychiatry.

[bib0008] Cloitre M., Cohen L.R., Ortigo K.M., Jackson C., Koenen K.C. (2020).

[bib0009] Cloitre M., Stovall-McClough K.C., Nooner K., Zorbas P., Cherry S., Jackson C.L., Petkova E. (2010). Treatment for PTSD related to childhood abuse: A randomized controlled trial. American Journal of Psychiatry.

[bib0010] Corrigan J.P., Fitzpatrick M., Hanna D., Dyer K.F. (2020). Evaluating the effectiveness of phase-oriented treatment models for PTSD—A meta-analysis. Traumatology.

[bib0011] Cruz D., Lichten M., Berg K., George P. (2022). Developmental trauma: Conceptual framework, associated risks and comorbidities, and evaluation and treatment. Frontiers in Psychiatry.

[bib0012] Dalgleish T., Black M., Johnston D., Bevan A. (2020). Transdiagnostic approaches to mental health problems: Current status and future directions. Journal of Consulting and Clinical Psychology.

[bib0013] De Prisco M., Oliva V., Fico G., Radua J., Grande I., Roberto N., Murru A. (2023). Emotion dysregulation in bipolar disorder compared to other mental illnesses: A systematic review and meta-analysis. Psychological Medicine.

[bib0014] Dieujuste N., Petri J.M., Mekawi Y., Lathan E.C., Carter S., Bradley B., Powers A. (2025). Investigating associations between emotion dysregulation and DSM-5 posttraumatic stress disorder (PTSD) using network analysis. Journal of Affective Disorders.

[bib0015] Djelantik A.M.J., Robinaugh D.J., Kleber R.J., Smid G.E., Boelen P.A. (2020). Symptomatology following loss and trauma: Latent class and network analyses of prolonged grief disorder, posttraumatic stress disorder, and depression in a treatment-seeking trauma-exposed sample. Depression and Anxiety.

[bib0016] Edwards E.R. (2022). Posttraumatic stress and alexithymia: A meta-analysis of presentation and severity. Psychological Trauma: Theory, Research, Practice, and Policy.

[bib0017] Epskamp S., Borsboom D., Fried E.I. (2018). Estimating psychological networks and their accuracy: A tutorial paper. Behavior Research Methods.

[bib0018] Epskamp S., Fried E.I. (2018). A tutorial on regularized partial correlation networks. Psychological Methods.

[bib0019] Fan L., Kang T. (2025). Early childhood trauma and its long-term impact on cognitive and emotional development: A systematic review and meta-analysis. Annals of Medicine.

[bib0020] First M.B., Spitzer R.L., Gibbon M., Williams J.B.W. (1996).

[bib0021] Ferguson S.L., G. Moore E.W., Hull D.M (2020). Finding latent groups in observed data: A primer on latent profile analysis in Mplus for applied researchers. International Journal of Behavioral Development.

[bib0022] Ford J.D. (2025). Dissociation and emotion dysregulation: New findings and nuances. Journal of Trauma & Dissociation.

[bib0023] Garofalo C., Neumann C.S., Kosson D.S., Velotti P. (2020). Psychopathy and emotion dysregulation: More than meets the eye. Psychiatry Research.

[bib0024] Gibson L.E., Alloy L.B., Ellman L.M. (2016). Trauma and the psychosis spectrum: A review of symptom specificity and explanatory mechanisms. Clinical Psychology Review.

[bib0025] Goh E.X.N., Tan C.M., Oon L.K., Subramaniam M., Tang C., Verma S., Jianlin L. (2026). Systematic review: Are complex posttraumatic stress disorder and dissociation associated with early-onset psychosis?. Journal of the American Academy of Child & Adolescent Psychiatry.

[bib0026] Goodman L.A., Corcoran C., Turner K., Yuan N., Green B.L. (1998). Assessing traumatic event exposure: General issues and preliminary findings for the stressful life events screening questionnaire. Journal of Traumatic Stress.

[bib0027] Gratz K.L., Roemer L. (2010). Multidimensional assessment of emotion regulation and dysregulation: Development, factor structure, and initial validation of the difficulties in emotion regulation scale. Journal of Psychopathology and Behavioral Assessment.

[bib0028] Gross J.J., John O.P. (2003). Individual differences in two emotion regulation processes: Implications for affect, relationships, and well-being. Journal of Personality and Social Psychology.

[bib0029] Grubaugh A.L., Zinzow H.M., Paul L., Egede L.E., Frueh B.C. (2011). Trauma exposure and posttraumatic stress disorder in adults with severe mental illness: A critical review. Clinical Psychology Review.

[bib0030] Hales G.K., Saribaz Z.E., Debowska A., Rowe R. (2023). Links of adversity in childhood with mental and physical health outcomes: A systematic review of longitudinal mediating and moderating mechanisms. Trauma, Violence, & Abuse.

[bib0031] Halstead S., Cao C., Mohr G.H., Ebdrup B.H., Pillinger T., McCutcheon R.A., Warren N. (2024). Prevalence of multimorbidity in people with and without severe mental illness: A systematic review and meta-analysis. The Lancet Psychiatry.

[bib0032] Hansen M., Ross J., Armour C. (2017). Evidence of the dissociative PTSD subtype: A systematic literature review of latent class and profile analytic studies of PTSD. Journal of Affective Disorders.

[bib0033] Hardy A. (2017). Pathways from trauma to psychotic experiences: A theoretically informed model of posttraumatic stress in psychosis. Frontiers in Psychology.

[bib0034] Haws J.K., Brockdorf A.N., Gratz K.L., Messman T.L., Tull M.T., DiLillo D. (2022). Examining the associations between PTSD symptoms and aspects of emotion dysregulation through network analysis. Journal of Anxiety Disorders.

[bib0035] Hochstrasser L., Studerus E., Riecher-Rössler A., Schimmelmann B.G., Lambert M., Lang U.E., Huber C.G. (2022). Latent state-trait structure of BPRS subscales in clinical high-risk state and first episode psychosis. Scientific Reports.

[bib0036] Hopko D.R., Reas D.L., Beck J.G., Stanley M.A., Wetherell J.L., Novy D.M., Averill P.M. (2003). Assessing worry in older adults: Confirmatory factor analysis of the Penn State Worry Questionnaire and psychometric properties of an abbreviated model. Psychological Assessment.

[bib0037] Hobfoll S.E. (2011). Conservation of resources theory: Its implication for stress, health, and resilience. The Oxford Handbook of Stress, Health, and Coping.

[bib0038] Hundt N.E., Ecker A.H., Thompson K., Helm A., Smith T.L., Stanley M.A., Cully J.A. (2020). It didn't fit for me:” A qualitative examination of dropout from prolonged exposure and cognitive processing therapy in veterans. Psychological Services.

[bib0039] Isvoranu A.M., van Borkulo C.D., Boyette L.L., Wigman J.T., Vinkers C.H., Borsboom D., Group Investigators (2017). A network approach to psychosis: Pathways between childhood trauma and psychotic symptoms. Schizophrenia Bulletin.

[bib0040] Isvoranu A.M., Epskamp S., Cheung M.W.L. (2021). Network models of posttraumatic stress disorder: A meta-analysis. Journal of Abnormal Psychology.

[bib0041] Isvoranu A.M., Epskamp S. (2023). Which estimation method to choose in network psychometrics? Deriving guidelines for applied researchers. Psychological Methods.

[bib0042] Iyadurai L., Visser R.M., Lau-Zhu A., Porcheret K., Horsch A., Holmes E.A., James E.L. (2019). Intrusive memories of trauma: A target for research bridging cognitive science and its clinical application. Clinical Psychology Review.

[bib0043] Jannini T.B., Daniele G., Rossi R., Niolu C., Di Lorenzo G. (2025). Emotional dysregulation in complex Post-traumatic Stress disorder: A narrative review. Journal of Psychopathology.

[bib0044] Kaufman E.A., Xia M., Fosco G., Yaptangco M., Skidmore C.R., Crowell S.E. (2016). The difficulties in emotion regulation scale short form (DERS-SF): Validation and replication in adolescent and adult samples. Journal of Psychopathology and Behavioral Assessment.

[bib0045] Klein A.B., Dutra S.J., Bovin M.J., Keane T.M., Marx B.P. (2021). The role of negative affect in differentiating posttraumatic stress disorder, depression, and their comorbidity among United States veterans. Journal of Traumatic Stress.

[bib0046] Liu J., Tan C.M., Ho J.C., Tang C., Verma S., Subramaniam M. (2025). Dissociation as transdiagnostic mediator between trauma exposure and the post-traumatic stress disorder-psychosis symptom spectrum: A structural equation modelling meta-analysis. Journal of Affective Disorders.

[bib0047] Liu J., Teh W.L., Tan R.H.S., Chang S.S.H., Lau B.J., Chandwani N., Subramaniam M. (2024). Evaluating a maladaptive personality-informed model of social support and post-traumatic stress disorder. Journal of Affective Disorders.

[bib0048] Liu J., Tan R.H.S., Chang S.S.H., Teh W.L., Shahwan S., Lee Y.W., Subramaniam M. (2024). Pathological personality explains individual differences in global emotion dysregulation within the pathway between child maltreatment and severe depressive symptoms. Psychological Trauma: Theory, Research, Practice, and Policy.

[bib0049] Liu J., Mahendran R., Chong S.A., Subramaniam M. (2021). Elucidating the impact of childhood, adulthood, and cumulative lifetime trauma exposure on psychiatric symptoms in early schizophrenia spectrum disorders. Journal of Traumatic Stress.

[bib0050] Liu J., Chua J.J.X., Chong S.A., Subramaniam M., Mahendran R. (2020). The impact of emotion dysregulation on positive and negative symptoms in schizophrenia spectrum disorders: A systematic review. Journal of Clinical Psychology.

[bib0051] Liu J., Subramaniam M., Chong S.A., Mahendran R. (2020). Maladaptive cognitive emotion regulation strategies and positive symptoms in schizophrenia spectrum disorders: The mediating role of global emotion dysregulation. Clinical Psychology & Psychotherapy.

[bib0052] Liu J., Lim M.S.M., Ng B.T., Chong S.A., Subramaniam M., Mahendran R. (2020). Global emotion dysregulation and maladaptive cognitive emotion regulation strategies mediate the effects of severe trauma on depressive and positive symptoms in early non-affective psychosis. Schizophrenia Research.

[bib0053] Lukoff D., Liberman R.P., Nuechterlein K.H. (1986). Symptom monitoring in the rehabilitation of schizophrenic patients. Schizophrenia Bulletin.

[bib0054] Lunansky G., Bonanno G.A., Blanken T.F., van Borkulo C.D., Cramer A.O., Borsboom D. (2024). Bouncing back from life’s perturbations: Formalizing psychological resilience from a complex systems perspective. Psychological Review.

[bib0055] Lynch S.J., Sunderland M., Newton N.C., Chapman C. (2021). A systematic review of transdiagnostic risk and protective factors for general and specific psychopathology in young people. Clinical Psychology Review.

[bib0056] Merrill R.M., Scott E.N., Sumsion L. (2026). Adverse childhood experiences impact unemployment and inability to work among adults in the United States. Frontiers in Psychology.

[bib0057] McKay M.T., Cannon M., Chambers D., Conroy R.M., Coughlan H., Dodd P., Clarke M.C. (2021). Childhood trauma and adult mental disorder: A systematic review and meta-analysis of longitudinal cohort studies. Acta Psychiatrica Scandinavica.

[bib0058] McLean C.P., Foa E.B. (2024). State of the science: Prolonged exposure therapy for the treatment of posttraumatic stress disorder. Journal of Traumatic Stress.

[bib0059] Miu A.C., Szentagotai-Tatar A., Balazsi R., Nechita D., Bunea I., Pollak S.D. (2022). Emotion regulation as mediator between childhood adversity and psychopathology: A meta-analysis. Clinical Psychology Review.

[bib0060] Naragon-Gainey K., McMahon T.P., Chacko T.P. (2017). The structure of common emotion regulation strategies: A meta-analytic examination. Psychological Bulletin.

[bib0061] Nehler K.J., Schultze M. (2024). Simulation-based performance evaluation of missing data handling in network analysis. Multivariate Behavioral Research.

[bib0062] Nvo-Fernandez M., Miño-Reyes V., Serrano C., Acosta-Antognoni H., Salas F., Wiedeman C.V., Leiva-Bianchi M. (2025). What is the impact of unemployment as an adverse experience? Post-traumatic stress disorder and complex post-traumatic stress disorder: A meta-analysis. International Journal of Environmental Research and Public Health.

[bib0063] Pugach C.P., Wisco B.E. (2023). Emotion regulation repertoires in trauma-exposed college students: Associations with PTSD symptoms, emotional awareness, and emotional clarity. Psychological Trauma: Theory, Research, Practice, and Policy.

[bib0064] Qiu L.S., Liddell B.J., Wong J., Li J.H., Lies J., Lau W., Jobson L. (2025). Cultural influences on emotion regulation and affective outcomes among trauma survivors: An ecological momentary assessment study. Journal of Affective Disorders.

[bib0065] Resick P.A., LoSavio S.T., Monson C.M., Kaysen D.L., Wachen J.S., Galovski T.E., Chard K.M. (2024). State of the science of cognitive processing therapy. Behavior Therapy.

[bib0066] Rnic K., Santee A.C., Hoffmeister J.A., Liu H., Chang K.K., Chen R.X., LeMoult J. (2023). The vicious cycle of psychopathology and stressful life events: A meta-analytic review testing the stress generation model. Psychological Bulletin.

[bib0067] Roach E.L., Haft S.L., Huang J., Zhou Q. (2023). Systematic review: The association between race-related stress and trauma and emotion dysregulation in youth of color. Journal of the American Academy of Child & Adolescent Psychiatry.

[bib0068] Schaumburg L.M., Nimtz C. (2026). Emotion dysregulation accounts for psychopathology associated with emotional abuse but not neglect: A systematic review of mediation studies. Child Abuse & Neglect.

[bib0069] See C.C., Tan J.M., Tan V.S., Sündermann O. (2022). A systematic review on the links between emotion regulation difficulties and obsessive-compulsive disorder. Journal of Psychiatric Research.

[bib0070] Seligowski A.V., Lee D.J., Bardeen J.R., Orcutt H.K. (2015). Emotion regulation and posttraumatic stress symptoms: A meta-analysis. Cognitive Behaviour Therapy.

[bib0071] Sideli L., Murray R.M., Schimmenti A., Corso M., La Barbera D., Trotta A., Fisher H.L. (2020). Childhood adversity and psychosis: A systematic review of bio-psycho-social mediators and moderators. Psychological Medicine.

[bib0072] Simpson L.E., Kumar S.A., Brockdorf A.N., Brock R.L., Messman T.L., Gratz K.L., DiLillo D. (2025). The cumulative impact of recurrent experiences of intimate partner violence on emotion dysregulation: A longitudinal investigation. Journal of Interpersonal Violence.

[bib0073] Stanton K., McDonnell C.G., Hayden E.P., Watson D. (2020). Transdiagnostic approaches to psychopathology measurement: Recommendations for measure selection, data analysis, and participant recruitment. Journal of Abnormal Psychology.

[bib0074] Subramaniam M., Abdin E., Vaingankar J.A., Shafie S., Chua B.Y., Sambasivam R., Chong S.A. (2020). Tracking the mental health of a nation: Prevalence and correlates of mental disorders in the second Singapore mental health study. Epidemiology and Psychiatric Sciences.

[bib0075] Thornton A., Gardner K.J., Graham-Kevan N., Parkinson J., Rynne R., Slankauskas N., Holland O. (2025). The relationship between adverse childhood experiences, emotion dysregulation and personality disorder traits: A systematic review and mediation analysis. Journal of Affective Disorders.

[bib0076] Treynor W., Gonzalez R., Nolen-Hoeksema S. (2003). Rumination reconsidered: A psychometric analysis. Cognitive Therapy and Research.

[bib0077] Tull M.T., Vidaña A.G., Betts J.E. (2020). Emotion in posttraumatic stress disorder.

[bib0078] van Minnen A., Harned M.S., Zoellner L., Mills K. (2012). Examining potential contraindications for prolonged exposure therapy for PTSD. European Journal of Psychotraumatology.

[bib0079] Virgolino A., Costa J., Santos O., Pereira M.E., Antunes R., Ambrósio S., Vaz Carneiro A. (2022). Lost in transition: A systematic review of the association between unemployment and mental health. Journal of Mental Health.

[bib0080] Wang J., Wang X. (2020).

[bib0081] Wang M., Fang J., Hu X., Cai T., Wu F., Lin Y. (2024). Chemotherapy-related symptoms in children with leukemia: Application of latent profile analysis and network analysis. Supportive Care in Cancer.

[bib0082] Wang S., Rodenbaugh M.M., Straud C., Weiss N.H., Contractor A.A. (2025). Exploring the co-occurrence of posttraumatic stress disorder symptoms and dysregulated positive emotion processes: A network analysis. Journal of Anxiety Disorders.

[bib0083] Wang S.K., Feng M., Fang Y., Lv L., Sun G.L., Yang S.L., Chen H.X. (2023). Psychological trauma, posttraumatic stress disorder and trauma-related depression: A mini-review. World Journal of Psychiatry.

[bib0084] Weathers F.W., Bovin M.J., Lee D.J., Sloan D.M., Schnurr P.P., Kaloupek D.G., Marx B.P. (2018). The clinician-administered PTSD Scale for DSM–5 (CAPS-5): Development and initial psychometric evaluation in military veterans. Psychological Assessment.

[bib0085] Weiss N.H., Contractor A.A., Raudales A.M., Greene T., Short N.A. (2020). Extending our understanding of the association between posttraumatic stress disorder and positive emotion dysregulation: A network analysis approach. Journal of Anxiety Disorders.

[bib0086] Wislocki K., Kratz H.E., Martin G., Becker-Haimes E.M. (2023). The relationship between trauma exposure and obsessive–compulsive disorder in youth: A systematic review. Child Psychiatry & Human Development.

[bib0087] Xue S., Lu A., Chen W., Li J., Ke X., An Y. (2024). A latent profile analysis and network analysis of anxiety and depression symptoms in Chinese widowed elderly. Journal of Affective Disorders.

[bib0088] Zhang C., Haim-Nachum S., Prasad N., Suarez-Jimenez B., Zilcha-Mano S., Lazarov A., Zhu X. (2025). PTSD subtypes and their underlying neural biomarkers: A systematic review. Psychological Medicine.

[bib0089] Zhang L., Wu X., Liu M., Liu A. (2025). Relationship between complex posttraumatic stress disorder symptoms and posttraumatic growth among adolescents and emerging adults with adverse childhood experiences. Psychological Trauma: Theory, Research, Practice, and Policy.

[bib0090] Zumstein N., Riese F. (2020). Defining severe and persistent mental illness – A pragmatic utility concept analysis. Frontiers in Psychiatry.

