# Supplementary material for: Synergising latent profile analysis and network analysis to elucidate the relationships among emotion dysregulation, post-traumatic stress disorder, dissociation, psychosis, and affective symptoms in individuals with trauma and severe mental illness
Source: Int J Clin Health Psychol. 2026 Aug 25;26(2):100714. doi: 10.1016/j.ijchp.2026.100714 (PMC13527492; doi:10.1016/j.ijchp.2026.100714)
Supplement: Supplementary file 1 [file mmc1.docx]

**Supplementary Figure Captions.**

Supplementary Figure 1. Centrality plot for node strength (Profile 1; N = 200).

Supplementary Figure 2. Centrality plot for node strength (Profile 2; N = 173).

Supplementary Figure 3. Centrality plot for bridge strength (Profile 2; N = 173).

Supplementary Figure 4. Centrality plot for node strength (Profile 3; N = 88).

Supplementary Figure 5. Centrality plot for bridge strength (Profile 3; N = 88).

Supplementary Figure 6. Centrality plot for node strength (Whole sample; N = 461).

Supplementary Figure 7. Centrality plot for bridge strength (Whole sample; N = 461).
